# Estradiol and progesterone from pregnancy to postpartum: a longitudinal latent class analysis

**DOI:** 10.3389/fgwh.2024.1428494

**Published:** 2024-10-09

**Authors:** Jelena Dukic, Alexandra Johann, Mirka Henninger, Ulrike Ehlert

**Affiliations:** ^1^ Clinical Psychology and Psychotherapy, Psychological Department, University of Zurich, Zurich, Switzerland; ^2^ Center for Statistics & Data Science, Faculty of Psychology, University of Basel, Basel, Switzerland; ^3^ Psychological Methods, Evaluation and Statistics, Psychological Department, University of Zurich, Zurich, Switzerland

**Keywords:** hormones, sex steroids, estradiol, progesterone, pregnancy, postpartum, peripartum, latent-class group analysis

## Abstract

**Introduction:**

During the peripartum, women undergo significant hormonal changes that are crucial for fetal development and a healthy pregnancy and postpartum period for mother and infant. Although several studies have determined healthy norm ranges of estradiol and progesterone, there are discrepancies among the reports, rendering it unclear which hormone levels are linked to adverse health outcomes. To account for the impact of sex steroid patterns on health outcomes in mothers and children, a longitudinal assessment of different parameters is needed.

**Materials and methods:**

We longitudinally assessed a cohort of 130 women over five months during pregnancy and postpartum. The women provided saliva samples and completed psychosocial questionnaires. Hormone analyses were conducted using enzyme-linked immunosorbent assay (ELISA). Different parameters of estradiol and progesterone were analyzed and evaluated in relation to psychometric variables. To examine the presence of heterogenous hormonal trajectories in the peripartum, we applied group-based trajectory modelling as a special case of latent-class group analysis.

**Results:**

Estradiol and progesterone levels rose towards the end of pregnancy and dropped sharply after birth, with considerable individual variation, particularly during pregnancy. However, their ratio remained stable. We identified three estradiol trajectory subgroups and two progesterone subgroups. Age influenced progesterone levels, with older pregnant women having higher levels than younger women. Anxiety and depressive symptoms had a predictive value for trajectories of specific subgroups of women. The study also revealed two distinct subgroups regarding the course of estradiol and progesterone fluctuations as well as their ratio.

**Conclusion:**

This study provides insights into the course and fluctuation of salivary estradiol and progesterone levels among healthy women during the peripartum period, highlighting significant variations in hormone levels but stability in their ratio during this time. The finding of distinct sex steroid courses in the peripartum is new and suggests the need for further research to explore their impact on health outcomes. Our preliminary results suggest that hormonal fluctuations at the end of pregnancy appear to be a normal occurrence and might even be a protective factor for associated psychological symptoms and sleep disturbances in women.

## Introduction

The peripartum represents an extraordinary endocrinological transition phase in a woman's life. It is only during pregnancy that an additional endocrinological organ is developed, the placenta. This organ performs all functions of the fetus's major organ systems as they differentiate and mature (1). Moreover, as part of the embryonic organism, the placenta also produces sex steroids (SS), which are important for the development of the fetus and the maintenance of pregnancy (2). The interaction and the feedforward mechanism between the placenta and the maternal hypothalamic-pituitary-gonadal (HPG) axis leads to a strong increase in estradiol (E2) and progesterone (P4) during pregnancy. With parturition and the accompanying detachment and expulsion of the placenta, SS levels drop rapidly (3). The female body is therefore exposed to strong hormonal changes during the rather short time period of the peripartum, which do not exist to this extent in other reproductive transition phases.

These peripartal hormonal changes are associated with numerous health outcomes in mothers and children. Increasing E2 levels during pregnancy are responsible for the growth and development of the fetus and its organs. Moreover, they stimulate the growth of the uterus and prepare the mammary glands for lactation. Increasing P4 levels are essential for maintaining pregnancy by preventing the uterus from contracting and triggering premature labor. They also prepare the breast tissue for milk production and suppress the maternal immune system to prevent rejection of the fetus. In the postpartum period, E2 and P4 are important for breastfeeding and uterine recovery (3–8). In sum, SS are crucial for the development of the fetus and for a healthy course of pregnancy and the postpartum period.

Furthermore, SS are not only relevant for a successful pregnancy and postnatal maternal well-being but can also be associated with negative health consequences for mothers and their children. These adverse outcomes include a variety of developmental, obstetric, autoimmune, neuronal, affective, and sleep pathologies and complications (9–17). In most cases, it is not yet clear how SS contribute to this wide range of pathologies. The complications and pathologies are usually associated with measurable deviations in SS from the normal range of each respective hormone. For instance, a recent study showed that women with severe anxiety in the third trimester of pregnancy have significantly higher E2 and lower P4 serum levels than women without anxiety (18). However, the various studies have yielded inconsistent findings with regard to the question of the level at which SS values are considered abnormal. As we recently revealed in a review, the SS concentrations reported across the different studies vary greatly at comparable gestational ages in the peripartum period (19). Consequently, a norm range of SS during pregnancy and postpartum is not yet clearly defined and it remains unclear which SS levels are associated with adverse health outcomes.

During pregnancy, maternal age is considered as an important determinant of maternal SS levels (20). In the first trimester of pregnancy, maternal age is found to be associated with lower E2 but higher P4 serum levels (21–23). A recent study reported that maternal age was also associated with lower E2 levels during the second trimester of pregnancy (24). To our knowledge, there are currently no data on influencing factors specifically referring to the postpartum period, although general determinants of steroid hormone variability have been established. Age is considered as the strongest predictor of SS concentrations, followed by body mass index (BMI) and race (25).

To better understand the relationship between SS levels in the peripartum period and health outcomes, knowledge of healthy SS release patterns is crucial. To achieve this, it is important to examine different aspects of physiological SS processes. First of all, secretion patterns of SS in peripartal women need to be longitudinally assessed in a highly standardized manner, to allow for conclusions about possible causal relationships and for the observation of intraindividual differences in SS courses. So far, only a limited number of studies have assessed SS longitudinally during pregnancy and postpartum in the same women, meaning that knowledge regarding the progression of SS trajectories during the entire peripartum period is limited (26, 27). Second, it is assumed that SS do not rise and fall continuously during the peripartum, but that these courses are accompanied by fluctuations in the corresponding hormones. SS fluctuations have already been observed during female puberty and the perimenopausal transition phase, with study findings suggesting that it is these hormonal fluctuations during puberty and perimenopause, rather than absolute hormone values, that are predictive of depressive symptoms, anxiety, negative affect, mood changes, anhedonia and cortisol levels (28–31). This pattern may be attributable to a general sensitivity to hormonal fluctuations during reproductive transitions over the life course in certain women (32). Given that high prevalence rates of psychopathological symptoms and illnesses are also reported in the peripartum period (33), studies need to investigate hormonal fluctuations in this phase as well, which to our knowledge has not yet been undertaken. As the hormonal status changes rapidly during this reproductive transition phase, SS measurements with large time intervals would not adequately detect fluctuations. Therefore, frequent measurements with short time intervals are necessary to detect the presence of SS fluctuations in the peripartum period. Third, E2 and P4 levels can each affect the impacts of the other hormone, with potential interactive effects on behavior and health outcomes (34). While E2 may stimulate the proliferation of P4 receptors (35), P4 inhibits the impact of E2 by reducing receptor densities (36). One potential approach to account for the joint impact of two interdependent hormones is to instead use a hormone ratio that reflects the simultaneous effects of both (34, 37). Following this approach, a recent study found that the peripartum ratio of allopregnanolone and P4 decreased more in women without anxiety than in women with anxiety (38). Even though the hormone ratio of E2 and P4 is a commonly used biomarker in fertility medicine and early pregnancy (39, 40), hormone ratios over the course of pregnancy and postpartum have scarcely been considered. In summary, there is a need to examine SS courses in the peripartum longitudinally and including potential hormonal fluctuations, and to draw on estimations of hormone ratios in addition to absolute values in order to adequately describe SS levels in the peripartum period and their relationship with health outcomes.

The present study aims to provide a detailed insight into the course of SS during the peripartum period by measuring E2 and P4 in saliva frequently and longitudinally over five months. The main goal is to describe the physiological course of salivary E2 and P4 in the peripartum period in a sample of physically healthy women. At the same time, interindividual differences in SS courses should be considered. For this purpose, we report absolute values of both E2 and P4, as well as their fluctuations and ratio, longitudinally in a large sample of pregnant and subsequently postpartum women. Saliva sampling is particularly suitable for this matter because it allows for numerous repeated measurements without great effort or risks for the participant (41). Thus, SS can be obtained repeatedly and frequently. A further aim of the present study is to examine the presence of heterogenous SS trajectories from pregnancy to postpartum. For this purpose, we apply latent class analysis to examine interindividual differences in the longitudinal course of SS in the peripartum period. With this approach, we seek to examine whether there are subgroups of women with distinct SS trajectories and whether these distinct trajectories can be explained by covariates such as psychological and physical symptoms. Ultimately, we hope that this will contribute to a better understanding and prevention of adverse health outcomes in mothers and children in the future.

## Materials and methods

The present study was conducted as an observational, longitudinal, single-center, national study. It was part of a large research project funded by the Swiss National Science Foundation, carried out at the Department of Clinical Psychology and Psychotherapy, University of Zurich. Physically healthy women were included in the study for a mean duration of 17 weeks per participant. Data collection took place between June 2019 and June 2021. Participants were included in the study during their third trimester of pregnancy, at 34–36 weeks of gestation, and were followed up until 8–12 weeks postpartum. One central aim of the larger project was to measure and analyze a variety of biopsychosocial factors associated with the female reproductive transition phase throughout pregnancy and postpartum. The present study represents a longitudinal investigation of salivary SS secretion patterns throughout all biological assessment points, namely at gestational weeks 34–36 and 40 and postpartum weeks 1, 4 and 8. Detailed information about the study design can be found elsewhere (42). This study was approved by the Ethics Committee of the Canton of Zurich (KEK-ZH-Nr. 2018–02357) and conducted according to the Declaration of Helsinki.

### Participants

Participants were recruited in the Greater Zurich area, Switzerland, through internet advertisements, social media (Facebook, Instagram), birth clinics, obstetricians, and antenatal classes. To participate, women needed to be currently pregnant, physically healthy, aged between 20 and 45 years and German-speaking. Besides mentally healthy women, we also included women with a history of depression and/or current depressive symptoms. To rule out other confounding mental health comorbidities, potential participants were screened by a member of the study team using the German version of the Structured Clinical Interview for DSM-IV (43) upon enrollment. To standardize hormonal influences, women with multifetal gestation or pregnancies achieved through assisted reproductive technology were excluded from the study. In addition, any medical complications [e.g., hypertension, diabetes mellitus, hyperemesis gravidarum, (pre-)eclampsia, suspected fetal growth restriction, fetal structural abnormalities] or medical conditions that might have affected ovarian function prior to pregnancy (e.g., polycystic ovary syndrome, endometriosis, breast cancer) led to study exclusion. Furthermore, women with a history of or current psychosis, bipolar disorder, posttraumatic stress disorder, eating disorders, and substance abuse or dependency were excluded. To eliminate any hormonal confounding variables, women with current intake of hormones (e.g., corticosteroids), diuretics, hypertensives, or vasodilators were excluded. The final exclusion criteria were treatment with psychotropic substances within the last three months prior to study inclusion, drug use and/or smoking, alcohol intake of more than one standard unit a day, pre-pregnancy BMI > 25 or <18, a protein-restricted diet, and/or extensive consumption of soy products. All participants provided informed consent prior to assessment.

**Figure 1:**
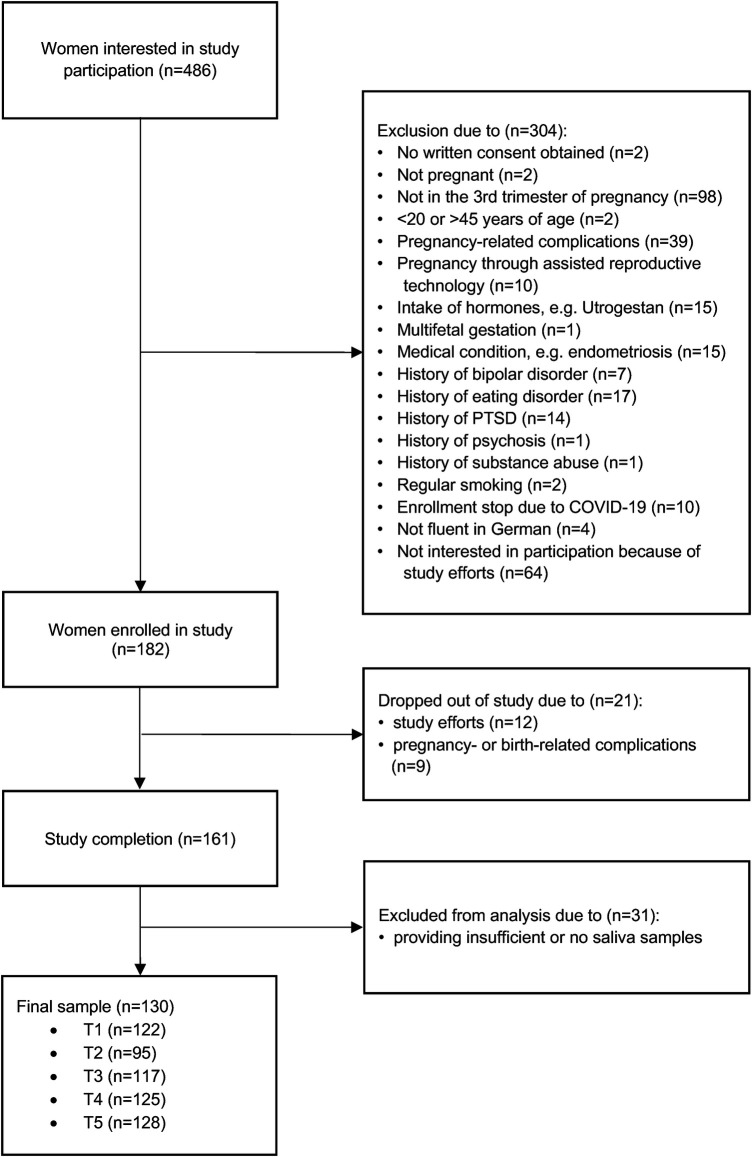
Flow diagram of participants recruited for this study. A total of 486 women were interested in participating in the study. Of these, 304 were excluded because they did not meet the inclusion criteria and 21 dropped out because they found the effort of participating in the study was too high or because of complications during pregnancy or after childbirth. A further 31 were excluded because they provided insufficient or no saliva samples, resulting in a final sample of 130 women.

We prospectively screened 486 women during the study period. A total of 182 women were included in the study and 161 women completed the study procedure. Of these, 130 ultimately provided sufficient saliva samples for the analysis of SS. The main reasons for dropout were preterm birth, complications during delivery, and the time expenditure associated with study participation. Women who dropped out of the study after enrolment were mostly Swiss, in a long-term relationship, still working, primiparous, and with a university degree. They were not in current psychiatric-psychotherapeutic care and most had no history of depression. Women who provided no or insufficient saliva samples mostly reported that they forgot to take the samples, were unable to produce enough saliva, or that the effort involved in providing saliva samples was too great. The flow diagram of participant selection is shown in Figure 1.

### Procedure

**Figure 2:**
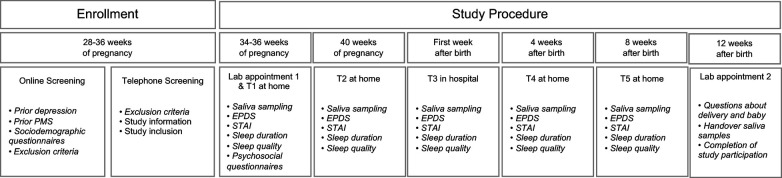
Study procedure. Women who were interested in participating in the study were screened for eligibility using an online questionnaire. Eligible women underwent a telephone interview to confirm the inclusion criteria and finalize enrolment. At 34–36 weeks of gestation, each participant was invited to our laboratory, where a strictly standardized protocol took place. Among multiple psychometric questionnaires, participants received instructions on saliva sampling, and were asked to independently collect saliva samples at home over the course of study participation (at 34–36 weeks of gestation, at 40 weeks of gestation, in the first week after birth, 4 weeks after birth, and 8 weeks after birth). Finally, the second lab appointment was scheduled around 8–12 weeks after giving birth. During this appointment, the same parameters as during the first lab appointment were re-assessed, birth-related information was included, and participants handed over their saliva samples. EPDS, Edinburgh Postnatal Depression Scale; STAI, State-Trait Anxiety Inventory; PMS, German PMS Inventory; MRS = Menopause Rating Scale-II.

Women who were interested in participating in the study were screened for eligibility using an online questionnaire. Eligible women who provided written consent then underwent a telephone interview to confirm the inclusion criteria and finalize enrolment. At 34–36 weeks of gestation, each participant was invited to our lab at the University of Zurich, where a strictly standardized protocol took place, starting between 08:00 and 09:00 a.m. Among multiple psychometric questionnaires and other biophysiological assessments, participants received instructions on saliva sampling verbally and in writing. They were then asked to independently collect saliva samples at home over the course of study participation, for a total of 52 time points. Finally, the second and last lab appointment was scheduled around 8–12 weeks after giving birth. During this appointment, the same parameters as during the first lab appointment were re-assessed and birth-related information was additionally included. An overview of the study procedure and assessment time points can be found in Figure 2. Detailed information about the study procedure can be found elsewhere (42).

### Salivary hormone assessment

A targeted total of 52 saliva samples per participant were collected under standardized conditions to determine E2 and P4 levels (pg/ml). As shown in Figure 2, saliva sampling occurred during five different measurement phases of study participation: on two successive days during gestational weeks 34–36 (T1), on two successive days during gestational week 40 (T2), on five successive days after giving birth (T3), on two successive days four weeks after giving birth (T4), and on two successive days eight weeks after giving birth (T5). On each collection day, saliva samples were taken four times daily: immediately after awakening, 30 and 45 min after awakening, and between 08:00 and 10:00 pm. These measurement time points were chosen to enable the additional assessment of diurnal cortisol levels and to ensure longitudinal and yet closely spaced analyses of endocrine courses. E2 levels were measured in a total of 5,142 samples and P4 levels were measured in a total of 4,937 samples, with data from 122 participants at T1, 95 participants at T2, 117 participants at T3, 125 participants at T4, and 128 participants at T5. Within the following analyses, the sample sizes vary on account of scattered missing data. For example, women who gave birth before 40 weeks of gestation were unable to provide saliva samples at T2. Similarly, some women did not provide saliva samples immediately after delivery or did not start saliva sampling on time at T3 (up to 48 h after delivery). These women were also excluded from the saliva analysis at T3.

The participants gathered saliva samples using the passive drool method in SaliCap sampling tubes with 2 ml capacity (IBL International GmbH, Hamburg, Germany). Participants were instructed to store the saliva samples in their own freezers after collection, until handing them over during the second lab appointment. All saliva samples were subsequently stored at −20°C at the laboratory of the University of Zurich. The hormone determinations were performed by Dresden LabService GmbH in Dresden, Germany. Hormone analyses were conducted using enzyme-linked immunosorbent assay (ELISA; IBL International GmbH, Hamburg, Germany, catalog number for E2: RE62141/RE62149, catalog number for P4: RE62021/RE62029). In the applied kits, the highest cross-reactivity for E2 was 14% with Estrone and for P4 1.8% with 17α-OH-Progesterone. The interassay coefficient of variability was 9.5% for E2% and 11% for P4. The intraassay coefficient of variability was <6% for both SS. The standard range was 2–64 pg/ml for E2 and 25–5,000 pg/ml for P4. In total, 20% of E2 samples and 0.9% of P4 samples were above the limit of detection, in particular during pregnancy at T1 and T2, when E2 and P4 levels are the highest ever observed in females. To deal with the values that were above the limit of detection, we used the method by Herbers et al. (44), who recommend imputing biomarker values beyond detection levels using a fitted lognormal distribution with the R package lnormimp (44). For E2, this affected *n* = 477 at T1, *n* = 562 at T2, *n* = 6 at T3, *n* = 3 at T4, *n* = 0 at T5, in total *n* = 1,048; for P4, it affected *n* = 28 at T1, *n* = 17 at T2, *n* = 0 at T3, *n* = 1 at T4, *n* = 0 at T5, in total *n* = 46).

### Self-report measures at study enrolment and completion

All relevant psychological variables in this study were assessed using validated German versions of self-report questionnaires. Online data were gathered using the Unipark platform (www.unipark.com/de, certificate ISO 27001). This reliable, BSI-certified data center fulfils the requirements of high data protection and safety in conformity with ISO 27001.

First, history of depression was assessed during the online screening, referring to a previous diagnosis of depression or self-report of prior depression, operationalized as yes/no. Second, to avoid missing cases, participants were asked about past depressive symptoms by presenting them with a list of the criteria for major depression according to the Diagnostic and Statistical Manual of Mental Disorders (5th ed.; DSM-5). If women confirmed three or more depressive symptoms over the course of at least two weeks in the past, they were included in the prior depression group.

The occurrence of premenstrual syndrome (PMS) in the past was also assessed during the online screening. For this purpose, participants were given a definition of PMS and were asked whether they had experienced it in the past, operationalized as yes/no.

During the first lab appointment, the current age and gravidity were assessed through an online questionnaire. Participants entered their current age numerically and selected the number of gravidities.

Upon study completion at the second lab appointment, participants were asked, using an online questionnaire, about the gestational week at delivery, delivery mode (vaginally or Caesarean section), gender of the baby (boy or girl) and how their infant had been fed up to that point (fully breastfed, mixed, or formula only). Hormonal withdrawal in the postpartum period has been linked to a variety of somatic symptoms (45). Due to the lack of an equivalent questionnaire for somatic symptoms postpartum, we applied the German version of the Menopause Rating Scale-II (MRS-II) to assess the occurrence of these somatic symptoms in the postpartum period (46, 47). The MRS-II is a highly reliable and validated instrument for the assessment of menopausal and quasi-menopausal symptoms, consisting of 11 self-report items (48).

### Self-report measures during study participation

In parallel with the saliva collections, participants completed online questionnaires on all days of saliva sampling. The questionnaires could be completed at any time of the day and assessed depressive and anxiety symptoms of the respective day and subjective sleep parameters of the previous night, among other variables.

Depressive symptoms during pregnancy and postpartum were assessed using the German version of the Edinburgh Postnatal Depression Scale (EPDS). The EPDS is a validated, widely used screening tool for postpartum depression. It consists of 10 self-report items and has shown satisfactory specificity and sensitivity (49). Participants completed the EPDS at the beginning of every saliva collection phase, for a total of five times.

Anxiety symptoms during pregnancy and postpartum were assessed using the short version of the State-Trait Anxiety Inventory (STAI-SKD) (50). The STAI-SKD is a short form of the widely used State-Trait Anxiety Inventory (STAI) (51) and has been demonstrated to be an efficient measure of state anxiety (52). Participants completed the STAI-SKD on every saliva collection day, for a total of 13 times.

On each day of saliva sampling, we also asked participants to subjectively rate their sleep quality and sleep duration of the previous night. Sleep quality could be rated as either very poor, poor, moderate, good, or very good, and sleep duration could be determined as 0–3, 4–6, 7–9, 10–11, or >12 h.

### Data analysis

#### Descriptive trajectories of E2 and P4 mean levels and fluctuations

To describe the trajectories of salivary SS in the peripartum period, we computed mean levels of E2 and P4 for each measurement day (13 in total) together with their log-transformation of ratio: log (P4/E2) = log(P4) - log(E2) (37, 44). Here, it is irrelevant whether the ratio is expressed as E2/P4 or P4/E2; of importance is that the ratio is logarithmically transformed to provide the same result (37). To describe the magnitude of hormonal fluctuations, we computed the mean absolute successive difference (MASD) (30, 53) for both SS and for each measurement phase (five in total).

#### Group-based trajectory modeling of E2 and P4 mean levels, their ratio and fluctuations

To describe interindividual differences in mean E2 and P4 levels, their ratio, and their fluctuations, we used group-based trajectory modelling (54–57). Group-based trajectory modelling is a special case of latent-class group analysis and can be used to describe and explain between-participant differences in growth trajectories over time. In our analysis, we used the R package lcmm (58). As person covariates, we used the aforementioned self-report measures assessed at study enrolment and study completion (history of depression, PMS in the past, age, gravidity, BMI in the pregnancy, delivery mode, gestational week at birth, gender of the baby). As time-varying covariates, we used the EPDS, STAI-SKD, subjective sleep quality, and sleep duration. Person covariates serve to explain latent group membership while time covariates serve to explain the relationship within participants on each measurement day or during each measurement phase. Missing values for covariates were imputed using the R package missForest (59), which uses a machine learning approach to impute missing values when variables are of different types (e.g., continuous and categorical data) or when variables are expected to be related in complex and non-linear ways.

#### Additional analyses in the peripartal phase

To gain more insights into the dynamics in the peripartal phase (Day 1 and Day 5 after birth), we investigated the relations of mean levels or fluctuations of E2 and P4 with history of depression, EPDS scores, and subjective sleep indicators (sleep duration and sleep quality) in mediation models using the lavaan package (60).

## Results

### Sample characteristics

Table 1 summarizes the sociodemographic characteristics of the sample. The mean maternal age was 33.3 years, 74% of the women were Swiss, 51% of the women were primiparous, 72% had a university degree, and 55% were married. Two women were smoking during the observed pregnancy and nine were drinking alcohol regularly. 30% of the participating women reported a previous depressive episode and 40% reported psychiatric illness in their family. 55% of the women delivered girls and 45% boys; 72% of the deliveries occurred vaginally and 28% through C-section. After delivery, 74% of the women were exclusively breastfeeding their babies, 19% used a combination of breastfeeding and formula-feeding, and 5% fed their children with formula only.

**Table 1:** Sociodemographic, psychosocial, and behavioral characteristics of the sample.

Variables	*N* (%) or mean [SD]
*N*	130
Age, years	33.3 [3.95], Min = 24, Max = 43
Primigravida, *n*	66 (51%)
Multigravida, *n*	64 (49%)
BMI at 34–36 weeks of gestation	26.3 [2.8], Min = 20.1, Max = 35.5
Nationality	96 Swiss (74%) 23 German (18%) 4 Austrian (3%) 19 others (15%)^a^
Education	19 vocational school (15%) 7 higher vocational school (5%) 5 general university entrance qualification (4%) 93 university degree (72%) 16 other (9%)^a^
Relationship status	56 unmarried (43%): • 1 single • 1 in a relationship but not cohabiting • 54 in a relationship and cohabiting 71 married (55%): • 1 married but not cohabiting • 70 married and cohabiting 3 divorced (2%): • 1 single • 2 in a new relationship but not cohabiting
Smoking in current pregnancy	128 never (98%) 2 up to two cigarettes per day (2%)
Alcohol intake in current pregnancy	121 never (93%) 8 up to two glasses per week (6%) 1 two to five glasses per week (1%)
Psychiatric illness in family	52 yes (40%): • 34 major depression • 5 bipolar disorder • 4 anxiety disorders • 2 obsessive-compulsive disorder • 3 eating disorders • 6 schizophrenia • 8 others/not specified 52 no (40%) 26 unknown (20%)
PPD of a female family member	19 yes (15%): 111 no (85%)
Prior depressive episode	39 yes (30%): • 6 during pregnancy • 6 during postpartum • 32 during other life phases^a^ 91 no (70%)
Prior PMS symptoms	12 yes (9%) 118 no (91%)
Pregnancy planned	102 yes (78%) 28 no (22%)
Delivery mode	93 vaginal (72%) 37 C-section (28%)
Gender of the baby	71 girls (55%) 59 boys (45%)
Baby nutrition	96 breastfeeding only (74%) 24 combined (19%) 7 formula only (5%) 3 not specified (2%)

^a^ Multiple answers possible, BMI, body mass index; PMS, premenstrual syndrome; PPD, peripartum depression.

### Trajectories of E2 and P4 in the peripartum

**Figure 3:**
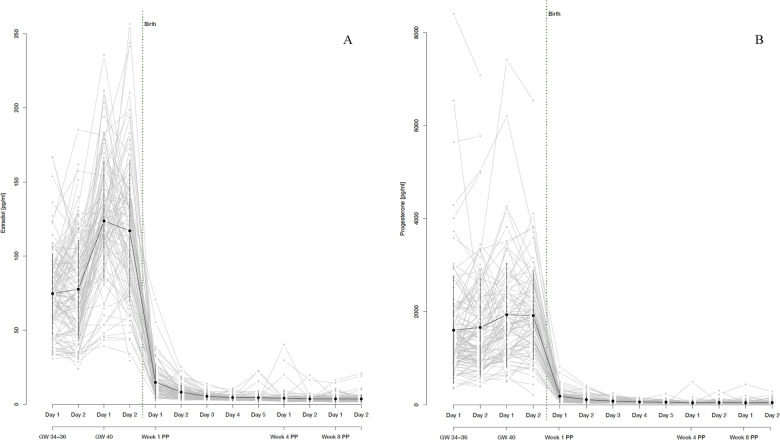
**(A)** salivary estradiol and **(B)** progesterone levels during pregnancy and postpartum. The grey dots and lines represent the mean and progressions of individual participants’ hormone levels for the respective sex steroid and time point. The black dot indicates the mean and the black line the standard deviation of all participants for the respective time point. Mean values and standard deviations for E2: GW 34-36, day 1 74.66 pg/ml (27.02), GW 34–36, day 2 77.66 pg/ml (32.84), GW 40, day 1 123.68 pg/ml (40.44), GW 40, day 2 117.05 pg/ml (46.96), Week 1 PP, day 1 14.88 pg/ml (10.95), Week 1 PP, day 2 8.27 pg/ml (4.62), Week 1 PP, day 3 5.44 pg/ml (2.03), Week 1 PP, day 4 4.59 pg/ml (1.72), Week 1 PP, day 5 4.54 pg/ml (3.19), Week 4 PP, day 1 4.19 pg/ml (4.46), Week 4 PP, day 2 3.75 pg/ml (2.14), Week 8 PP, day 1 3.73 pg/ml (2.13), Week 8 PP, day 2 3.69 pg/ml (2.46). Mean values and standard deviations for P4: GW 34–36, day 1 1,599.95 pg/ml (1,138.52), GW 34–36, day 2 1,658.98 pg/ml (1,051.58), GW 40, day 1 1,932.16 pg/ml (1,106.90), GW 40, day 2 1,914.03 pg/ml (935.67), Week 1 PP, day 1 184.06 pg/ml (133.76), Week 1 PP, day 2 109.59 pg/ml (76.20), Week 1 PP, day 3 76.87 pg/ml (45.67), Week 1 PP, day 4 59.92 pg/ml (29.18), Week 1 PP, day 5 55.25 pg/ml (32.02), Week 4 PP, day 1 43.53 pg/ml (44.85), Week 4 PP, day 2 46.99 pg/ml (36.39), Week 8 PP, day 1 43.77 pg/ml (43.16), Week 8 PP, day 2 45.04 pg/ml (36.42). GW, gestational week; PP, postpartum.

**Figure 4:**
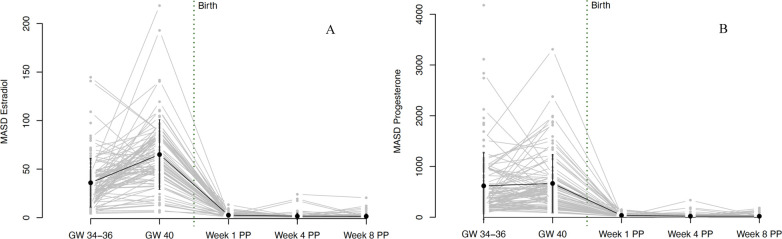
Fluctuations in **(A)** estradiol and **(B)** progesterone levels during pregnancy and postpartum. Fluctuations were operationalized as mean absolute successive difference (MASD). The grey dots and lines represent the mean and progression of individual participants’ fluctuations (operationalized as MASD) for the respective sex steroid. The black dot indicates the mean and the black line the standard deviation of all participants for the respective time point. Mean MASD values of fluctuations and the respective standard deviations for E2: GW 34–36 35.97 (25.1), GW 40 65.04 (35.82), Week 1 PP 2.6 (2.14), Week 4 PP 1.55 (3.16), Week 8 PP 1.41 (2.42). Mean values of fluctuations and the respective standard deviations for P4: GW 34–36 618.8 (655.01), GW 40 666.57 (568.09), Week 1 PP 36.89 (31.28), Week 4 PP 21.49 (38.99), Week 8 PP 20.72 (31.2). GW, gestational week; MASD, mean absolute successive difference; PP, postpartum.

During the third trimester of pregnancy, both E2 and P4 levels were extremely high and continued to increase until 40 weeks of gestation (see Figure 3). At the same time, both hormones showed strong interindividual variations, as well as intraindividual fluctuations even between successive assessment days (see Figure 4). With birth, both hormones dropped massively in all women and continued to decrease during the first postpartum days. On days 4 and 5 of the first week postpartum, a few women already showed increases in hormone concentrations again. These fluctuations continued through weeks 4 and 8 postpartum, with most women remaining in very low hormone ranges. The mean values of the measured E2 and P4 concentrations for each assessment day of the peripartum are listed in Table 2 and graphically depicted in Figure 3.

**Table 2:** Mean estradiol, progesterone levels, the respective fluctuations and the ratio of the hormones [log (P4/E2)] by time point (pg/ml).

		Mean of Estradiol (SD)	Mean of Progesterone (SD)	Mean of Estradiol MASD^a^ (SD)	Mean of Progesterone MASD^a^ (SD)	Mean of Hormone Ratio (SD)
Timepoint of sex steroid assessment						
Gestational week 34–36	Day 1	74.66 (27.02)	1,599.95 (1,138.52)	35.97 (25.1)	618.8 (655.01)	2.96 (0.56)
	Day 2	77.66 (32.84)	1,658.98 (1,051.58)			3.00 (0.58)
Gestational week 40	Day 1	123.68 (40.44)	1,932.16 (1,106.90)	65.04 (35.82)	666.57 (568.09)	2.70 (0.51)
	Day 2	117.05 (46.96)	1,914.03 (935.67)			2.78 (0.51)
Week 1 postpartum	Day 1	14.88 (10.95)	184.06 (133.76)	2.6 (2.14)	36.89 (31.28)	2.53 (0.47)
	Day 2	8.27 (4.62)	109.59 (76.20)			2.53 (0.54)
	Day 3	5.44 (2.03)	76.87 (45.67)			2.55 (0.50)
	Day 4	4.59 (1.72)	59.92 (29.18)			2.52 (0.40)
	Day 5	4.54 (3.19)	55.25 (32.02)			2.46 (0.56)
Week 4 postpartum	Day 1	4.19 (4.46)	43.53 (44.85)	1.55 (3.16)	21.49 (38.99	2.36 (0.50)
	Day 2	3.75 (2.14)	46.99 (36.39)			2.43 (0.46)
Week 8 postpartum	Day 1	3.73 (2.13)	43.77 (43.16)	1.41 (2.42)	20.72 (31.2)	2.38 (0.57)
	Day 2	3.69 (2.46)	45.04 (36.42)			2.39 (0.61)

^a^ MASD is an estimate of the fluctuations in the respective sex steroid, MASD, mean absolute successive difference.

**Figure 5:**
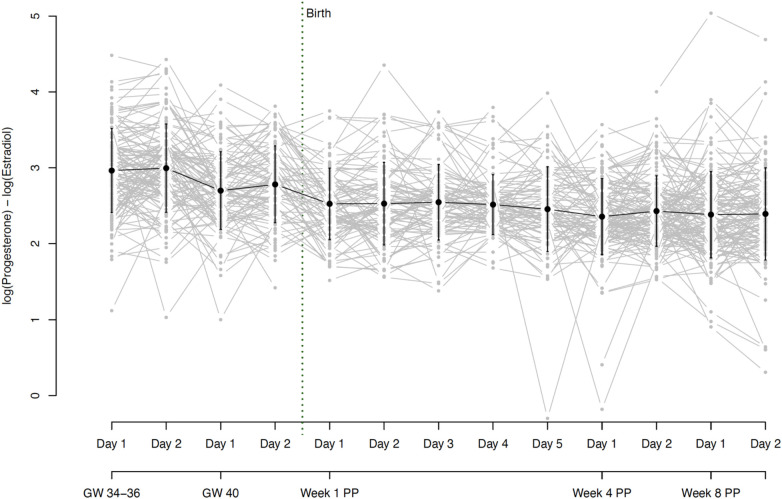
Hormone ratio of estradiol and progesterone (Log (P4/E2) = log (P4) - log (E2)) based on daily mean hormone levels. The grey dots and lines represent the individual participants’ hormone ratio for the respective time point. The black dot indicates the mean and the black line the standard deviation of all participants for the respective time point. Mean ratios and standard deviations: GW 34–36, day 1 2.96 (0.56), GW 34–36, day 2 3.00 (0.58), GW 40, day 1 2.70 (0.51), GW 40, day 2 2.78 (0.51), Week 1 PP, day 1 2.53 (0.47), Week 1 PP, day 2 2.53 (0.54), Week 1 PP, day 3 2.55 (0.50), Week 1 PP, day 4 2.52 (0.40), Week 1 PP, day 5 2.46 (0.56), Week 4 PP, day 1 2.36 (0.50), Week 4 PP, day 2 2.43 (0.46), Week 8 PP, day 1 2.38 (0.57), Week 8 PP, day 2 2.39 (0.61). GW, gestational week; PP, postpartum.

On average, the hormone ratio demonstrated a stable course over the entire peripartum period, with mean ratios between 2.4 and 3.0. Here too, however, there were strong interindividual as well as intraindividual differences over the whole peripartum period. The hormone ratio course is shown in Figure 5 and the mean values of the hormone ratio are listed in Table 2.

Both E2 and P4 fluctuated strongly during pregnancy. While the fluctuations in E2 increased significantly from 34 to 36 to 40 weeks of gestation, fluctuations in P4 remained constantly high during this phase. The fluctuations decreased massively with the drop in hormones after birth but then increased again somewhat, especially in E2 from the 4th week postpartum. Again, strong interindividual differences were evident, especially during pregnancy. The courses of hormonal fluctuations measured using the MASD are shown in Figure 4.

For all results reported so far, data within detection limits only (without imputed data using the method proposed by Herbers et al. (44) are shown in Supplementary Figures S1–S3. The trajectories in Figures 3–5 are based on the hormone values for which values above the detection limit were imputed according to the Herbers method (44). In the appendix, we also present the trajectories without these imputed values, thus with only the values that were within the detection limits. The trajectories look very similar in both cases but are presented in both ways for reasons of completeness.

### Group-based trajectory analysis of hormonal courses

**Figure 6:**
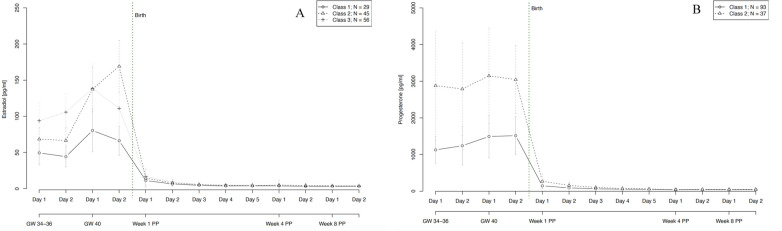
Trajectory groups according to the best-fitting prediction model of **(A)** estradiol and (**B)** progesterone courses during pregnancy and postpartum. The best-fitting model for estradiol **(A)** identified three significantly distinct trajectory groups: class 1 (⦵, *n* = 29, 22% of women), class 2 (∆, *n* = 45, 35% of women) and class 3 (+, *n* = 56, 43% of women). The best-fitting model for progesterone **(B)** identified two significantly distinct trajectory groups: class 1 (⦵, *n* = 93, 72% of women) and class 2 (∆, *n* = 37, 28% of women). GW, gestational week; PP, postpartum.

Our final model for E2 identified three trajectory groups. These groups showed significantly distinct courses of E2 levels during pregnancy and also during the first days after birth, as graphically represented in Figure 6A. The first trajectory group (*n* = 29, 22% of women) had the lowest E2 levels during pregnancy but also in the first days postpartum. The second trajectory group (*n* = 45, 35% of women) showed somewhat higher E2 levels during pregnancy and the strongest increase in E2 until the end of pregnancy, reaching up to 169 pg/ml shortly before giving birth. The third trajectory group (*n* = 56, 43% of women) is marked by the highest E2 values at gestational weeks 34–36 and a beginning decline in E2 levels in gestational week 40. The model showed no significant person covariates distinguishing the groups. Over time, E2 levels in the third group were significantly negatively associated with STAI scores (*W* = −0.619, *p* = 0.021).

Our final model for P4 identified two significantly distinct trajectory groups. In both groups, P4 levels remained relatively stable over the course of pregnancy, with only a slight increase towards gestational week 40. Again, the two groups differed mainly in their P4 levels during pregnancy and also in the first days after birth. The first group (*n* = 93, 72% of women) had noticeably lower levels of P4 especially during pregnancy compared to the second group (*n* = 37, 28% of women). Age significantly increased the likelihood of belonging to the first group with lower levels of P4 (*W* = 0.160, *p* = 0.019). Over time, P4 levels in the second group showed a significant positive association with EPDS scores (*W* = 0.190, *p* = 0.004) and a significant negative association with STAI scores (*W* = −0.312, *p* = 0.010).

**Figure 7:**
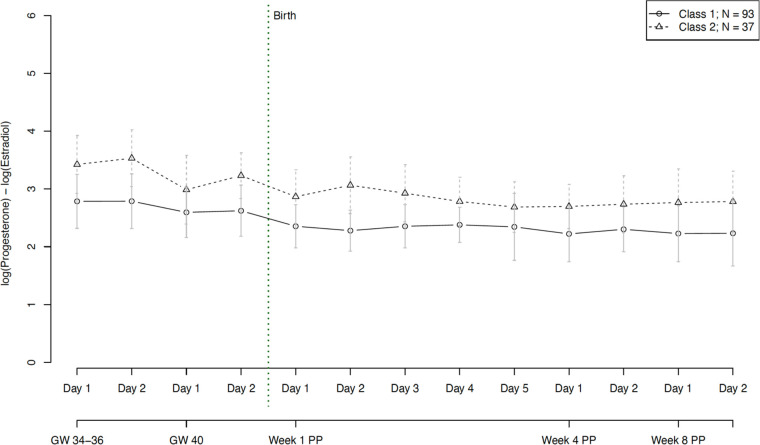
Trajectory groups according to the best-fitting prediction model of the hormone ratio of estradiol and progesterone (Log (P4/E2)). The best-fitting model for the progesterone ratio identified two significantly distinct trajectory groups: class 1 (⦵, *n* = 93, 72% of women) and class 2 (∆, *n* = 37, 28% of women). GW, gestational week; PP, postpartum.

With regard to hormone ratios, our final model revealed two significantly distinct trajectory groups, depicted in Figure 7. Group 1 (*n* = 93, 72% of women) showed a significantly lower hormone ratio over the whole course of pregnancy and postpartum, while group 2 (*n* = 37, 28% of women) retained a constantly slightly higher ratio, but with a parallel course to group 1. The model showed no significant person covariates distinguishing the two groups. Over time, the hormone ratio of the second group was significantly positively associated with EPDS scores (*W* = 0.014, *p* = 0.036) and subjective sleep duration (*W* = 0.081, *p* = 0.004), while the hormone ratio of the first group was negatively associated with STAI scores (*W* = −0.018, *p* = 0.007).

**Figure 8:**
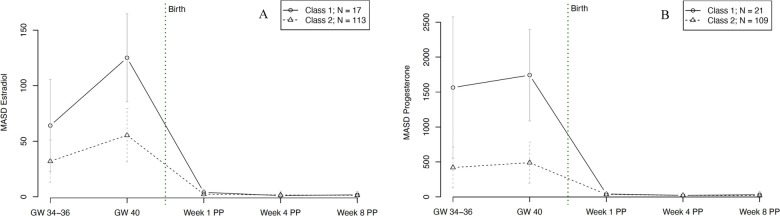
Trajectory groups according to the best-fitting prediction model of **(A)** fluctuations in estradiol and **(B)** fluctuations in progesterone levels during pregnancy and postpartum. Fluctuations were operationalized as mean absolute successive difference (MASD). The best-fitting model for estradiol fluctuations **(A)** identified two significantly distinct trajectory groups: class 1 (⦵, *n* = 17, 13% of women) and class 2 (∆, *n* = 113, 87% of women). The best-fitting model for progesterone fluctuations **(B)** identified two significantly distinct trajectory groups: class 1 (⦵, *n* = 21, 16% of women) and class 2 (∆, *n* = 109, 84% of women). GW, gestational week; MASD, mean absolute successive difference; PP, postpartum.

In terms of hormonal fluctuations, our final model revealed two significantly distinct trajectory groups for E2 fluctuations, graphically represented in Figure 8A. Group 1 (*n* = 17, 13% of women) showed strong E2 fluctuations at 34–36 weeks of gestation and even stronger fluctuations at 40 weeks of gestation. The E2 fluctuations in group 2 (*n* = 113, 87% of women) were significantly lower but followed the same pattern, with increasing fluctuations towards the end of pregnancy. In both groups, hormonal fluctuations in the postpartum were extremely low compared to antepartum. The model showed no significant person covariates distinguishing the two E2 fluctuation groups. Over time, the E2 fluctuations of the first group showed a significant positive association with subjective sleep duration (*W* = 6.469, *p* = 0.009).

For P4 fluctuations, our final model revealed two significantly distinct trajectory groups, as shown in Figure 8B. Group 1 (*n* = 21, 16% of women) showed significantly stronger fluctuations during pregnancy compared to group 2 (*n* = 109, 84% of women). After delivery, both groups consistently showed only very low fluctuations compared to during pregnancy. The model revealed no significant person covariates for the two P4 fluctuation groups. Over time, the P4 fluctuations of the first group were significantly negatively associated with STAI scores (*W* = −6.326, *p* = 0.011).

### Additional analyses

Hormone levels and fluctuations were significantly lower after birth (T3-T5) than before birth (T1-T2). To account for differences between the ante- and postpartum phases, we repeated the group-based trajectory analyses, but only for the postpartum measurement phases T3-T5 (without the measurement phases T1-T2). Here, we included additional postpartum person covariates, such as the infant's sex, birth weight and type of nutrition. In the postpartum phase (T3-T5), there were no latent classes, indicating that the means as well as the ratios and fluctuations after birth were homogeneous for all women.

**Figure 9:**
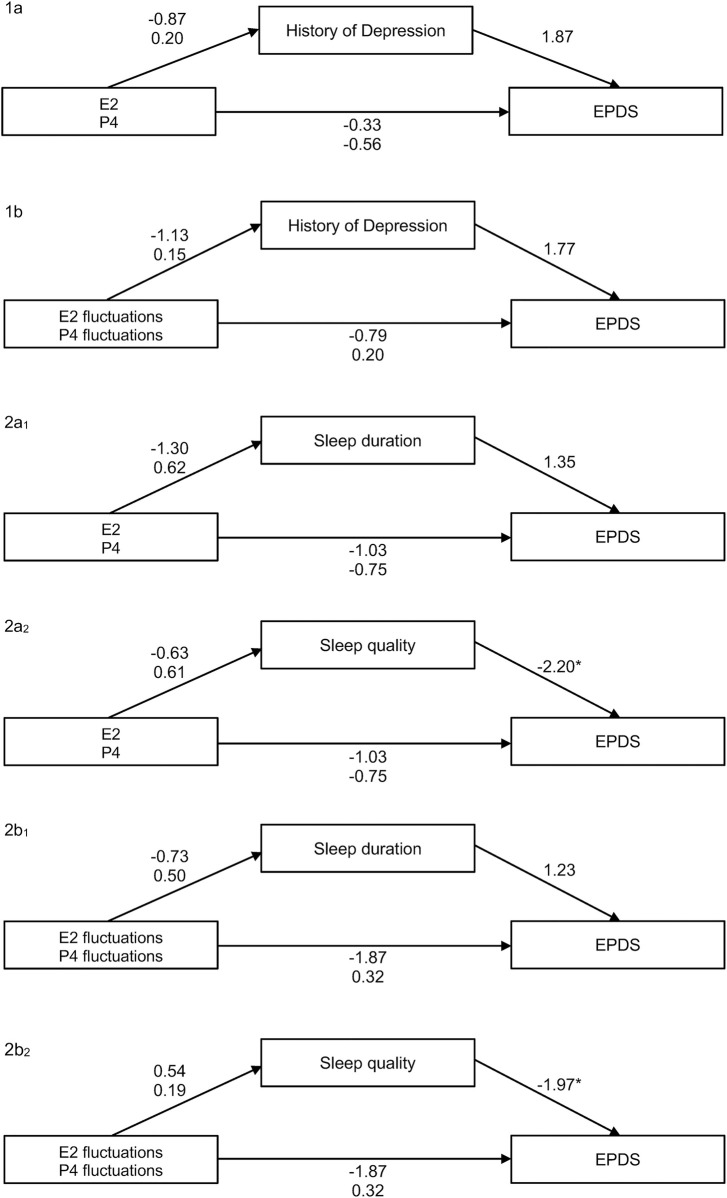
Overview of all mediation models tested: *1a* relationship between mean levels of E2 and P4 and the EPDS score, mediated by history of depression (NS). *1b* Relationship between fluctuations of E2 and P4 (via MASD) and the EPDS score, mediated by history of depression (NS). *2a_1_* Relationship between mean levels of E2 and P4 and the EPDS score, mediated by sleep duration (NS). *2a_2_* Relationship between fluctuations of E2 and P4 (*via* MASD) and the EPDS score, mediated by sleep quality (significant effect of subjective sleep quality on EPDS, *b* = −2.20, *p* = 0.028). *2b_1_* Relationship between fluctuations of E2 and P4 (via MASD) and the EPDS score, mediated by sleep duration (NS). *2b_2_* Relationship between fluctuations of E2 and P4 (via MASD) and the EPDS score, mediated by sleep quality (significant effect of subjective sleep quality on EPDS, *b* = −1.97, *p* = 0.049). EPDS, Edinburgh Postnatal Depression Scale; MASD, mean absolute successive difference.

Furthermore, we also estimated mediation models to analyze the relationship between mean levels or fluctuations of E2 and P4, history of depression, EPDS scores, and subjective sleep indicators (sleep duration and sleep quality). Details and metrics can be found in Figure 9. With the exception of one effect, all mediation models remained non-significant. However, there was a slightly significant negative association between subjective sleep quality and EPDS scores in two models (*b* = −2.20, *p* = 0.028 and *b* = −1.97, *p* = 0.049).

## Discussion

In the present study, saliva samples from E2 and P4 were collected and analyzed over a period of five months during the peripartum. Our results revealed increasingly high levels of both E2 and P4 towards the end of pregnancy and a massive drop in both hormones immediately after birth, with a persistent drop in the first few days postpartum. At the same time, these courses showed strong interindividual fluctuations in both E2 and P4, especially during pregnancy. Despite these fluctuations, the hormone ratio of the two SS remained very stable over the entire postpartum period. Since the courses of both hormones showed strong interindividual variation, we were able to demonstrate that these observed interindividual differences can be summarized into different subgroups. This finding is of particular importance, as it was previously assumed that the course of SS increases continuously during pregnancy in all women, before decreasing after delivery (3). Instead, our group-based trajectory analysis showed that E2 trajectories could be divided into three subgroups and that P4 trajectories could be divided into two subgroups. Age proved to be a significant person covariate, with older pregnant women showing a greater likelihood of having higher P4 levels compared to younger pregnant women. This result is in accordance with Toriola and colleagues, who found higher P4 levels in women above the age of 30 years in the first half of pregnancy compared to women below this age (20). However, it is in contrast to another study, which compared P4 levels in late pregnancy between women above and below 35 years of age and found no significant differences (61).

Besides the effect of age on P4, our findings did not reveal any relationship between person covariates and latent class membership. One reason for this might be that some of the resulting latent classes were relatively small in size (e.g., class 1 for E2 and E4 fluctuations, see Figure 8). These small latent class sizes result in larger standard deviations and a lower power to detect relationships with person covariates. Beyond that, time-varying covariates such as anxiety (for E2 and P4) and depressive symptoms (for P4) showed significant predictive value in the SS courses of specific subgroups of peripartum women, suggesting a correlation between SS levels and psychological symptoms in a subgroup of women. Over time, E2 levels of one subgroup were significantly negatively associated with anxiety scores, indicating that E2 could have protective anxiolytic effects during the peripartum in a subgroup of women. In turn, this may imply a role of underlying (epi-)genetic differences, rendering certain women more sensitive to differences in E2 levels than others. In the P4 courses, levels of one subgroup showed a significant positive association with depression scores and a significant negative association with anxiety scores. This finding reflects a potentially complex interplay of P4's effects in a specific subgroup of women, which may be attributable to individual variability in hormone sensitivity, receptor expression, or neurobiological pathways, leading to a protective effect against anxiety but a predisposition to depressive symptoms in certain women. The study also revealed two distinct subgroups with respect to fluctuations of E2 and P4 and their ratio over the course of the peripartum period. These subgroups showed differential associations with time-varying covariates such as anxiety, depression, and subjective sleep duration. As such, it appears that a specific hormone ratio may contribute to a depressive state while also leading to longer sleep durations, possibly as a coping mechanism or a symptom of depression. At the same time, the negative association with anxiety scores in the second group suggests that a different hormone ratio might have protective anxiolytic effects. In terms of hormonal fluctuations, our model revealed that E2 fluctuations of one subgroup showed a significant positive association with subjective sleep duration, suggesting that fluctuations in E2 levels might be linked to longer sleep duration, possibly through mechanisms involving sleep architecture modulation, circadian rhythm adjustments, neurotransmitter interactions, or affective stabilization. This finding highlights the complex role of E2 in sleep regulation and suggests that certain individuals may be particularly sensitive to these hormonal fluctuations in E2. Regarding P4 fluctuations, our model revealed that in one group fluctuations were significantly negatively associated with anxiety scores (*W* = −6.326, *p* = 0.011), suggesting that variability in P4 levels might be linked to reduced anxiety. This could be due to the anxiolytic effects of P4 metabolites like allopregnanolone, modulation of the stress response system, and individual sensitivity to hormonal changes. The findings highlight the complex role of P4 in emotion regulation and suggest that in certain individuals or contexts, fluctuations in this hormone can be beneficial for maintaining lower anxiety levels.

The presence of such strong hormonal fluctuations at the end of pregnancy raises questions about their function. For E2 and P4, we identified a small subgroup of women with significantly stronger fluctuations compared to all other pregnant women, especially shortly before labor. This group reported both longer subjective sleep duration and lower anxiety. More sleep and less anxiety, even at the end of pregnancy, are desirable for a healthy pregnancy. This is in line with the findings of Kundakovic and Rocks (62), who reported that SS fluctuations during the reproductive transition phases are usually physiological in women and may even be a protective factor for associated psychological states and sleep disturbances. However, this interpretation of the present results contradicts the hormone sensitivity hypothesis, according to which SS fluctuations are associated with a higher symptom burden during reproductive transition phases (63, 64). As fluctuations might also represent a means of adapting to changing circumstances, from a methodological perspective, fluctuations might be used to determine the new level at which values tend to stabilize (65).

Given that such individually fluctuating E2 and P4 values have also been described in the perimenopausal transition phase (29, 66) the occurrence of these fluctuations during both hormonal transition phases might be an indication of a comparable underlying mechanism. Although E2 fluctuations during perimenopause were associated with more depressive symptoms compared to our findings postpartum, the fluctuations during perimenopause were observed within time intervals of weeks or even months (29, 67, 68) and as our results show, fluctuations in SS concentrations happen within hours and days. Thus, the results from these two hormonal transition phases cannot be directly compared with each other. In the future, longitudinal studies assessing women repeatedly through different reproductive transition phases will be necessary to test the hormone sensitivity hypothesis. These studies should focus on the repeated occurrence of SS fluctuations in the same women and employ short- and long-term time intervals to identify different types of fluctuations. In addition, SS fluctuations should be studied in relation to higher or lower symptom burden.

Despite the fluctuations in E2 and P4, the ratio of the two hormones remained stable through the whole peripartum. This finding in physically healthy women may indicate that the joint impact of the two hormones might be important for a healthy course of the peripartum period. While previous research linked lower P4/E2 ratios to preterm birth, we are unable to confirm this finding as there were only a limited number of preterm cases in our sample (69). To the best of our knowledge, only one previous study has investigated ratios during the peripartum period and reported decreased P4/E2 ratios in women in active labor, indicating local hormonal changes during parturition (70). It is crucial to investigate hormone ratios in relation to perinatal outcomes, as existing research often focuses on the levels of individual hormones, without considering their ratio. In view of the consistent ratios observed in healthy women during pregnancy and postpartum in the present study, we recommend integrating maternal hormone ratios into future research on perinatal health outcomes.

E2 and P4 have effects on maternal health and fetal development during the peripartum period. Fluctuating SS levels affect neuronal gene expression, brain plasticity, and behavior in women (62, 71–76). Women with postpartum depression seem to have an altered sensitivity to E2 fluctuations in particular. Epigenetic mechanisms such as DNA methylation can alter SS receptor function, influencing SS levels and symptom occurrence (77, 78). In addition, women with perinatal depressive symptoms exhibit changes in gene expression, which have been linked to estrogen response, suggesting a potential influence of dysregulated cellular signaling on mood and/or peripartum depression risk (79, 80). Therefore, genetic and epigenetic changes in SS receptor functions may have explanatory value for the different courses of SS and associated symptoms in our study. Future research should explore genetic and epigenetic differences in SS receptor functioning in connection with SS levels and associated maternal parameters. SS may also impact fetal brain development, potentially leading to developmental disorders (19, 81). Fetal neurological development relies on placental E2 and P4 supply, rendering preterm birth a risk factor for cerebral complications (82). At the same time, levels in SS vary greatly during late pregnancy and are prone to strong fluctuations. As the consequences of this are still unknown, future research assessing SS fluctuations is essential. Besides E2 and P4, allopregnanolone has become increasingly important in the context of perinatal mental health in recent years. As a metabolite of P4, allopregnanolone increases during pregnancy and is synthesized by the gonadal and adrenal glands as well as by the placenta. It acts as neurosteroid and has numerous effects on maternal well-being as well as on fetal outcomes (83–86). Consequently, allopregnanolone, its fluctuations, and its ratios to SS should be analyzed in future longitudinal studies on factors influencing perinatal health outcomes.

To account for any effects in the immediate postpartum period, we examined this period separately in additional analyses. No distinct hormone trajectory groups could be identified, but we found a significant effect twice in the mediation models, suggesting a link between subjective sleep quality and depressive symptoms in this period. This finding is consistent with results from Okun and colleagues, who demonstrated that poor sleep quality across the first 17 weeks post-delivery increases the risk for recurrent depression, independent of hormonal changes (87). Nevertheless, it is not only the postnatal sleep quality that appears to be important here, but also the quality of sleep during pregnancy. Reduced sleep quality as early as the third trimester is associated with increased depression rates in the postpartum period (88, 89). At the same time, the subjective perception of sleep quality appears to have a stronger predictive value than objective sleep parameters in explaining postpartum mood (90). However, sleep quality is only one factor associated with maternal health. For a more profound understanding of peri- and postpartum maternal mental health, multidimensional approaches including a large number of known determinants are crucial (91). Some limitations need to be taken into consideration when interpreting the results of the present study. First, the ability to generalize our results is limited due to a relatively homogeneous study population. Second, psychometric characteristics were only captured via self-report and should be interpreted accordingly. Third, participants collected saliva samples depending on their respective time of awakening, potentially affecting endocrinological parameters through irregular sleep/wake cycles. Fourth, some women did not provide saliva samples in particular around the time of birth. Fifth, especially during pregnancy, numerous women reached SS values above the detection limits permitted by the analysis kit used. Although these were imputed using a well-established procedure, they nevertheless do not represent the exact values. In this context, it is important to mention the use of ELISA as the only analytical method to quantify SS. Comparisons with liquid chromatography–mass spectrometry (LC-MS/MS) have repeatedly shown that RIA and ELISA can be problematic for the quantification of SS metabolites, especially in very low and very high concentration ranges (92–96). At the same time, SS levels vary depending on the specimen used. While serum and plasma levels show values of a similar order of magnitude, saliva levels tend to be comparatively extremely low (19).

Besides these limitations, a major strength of our study lies in the longitudinal assessment of E2 and P4 at 52 time points over five months. This allowed for a detailed assessment of hormonal courses, fluctuations, and ratios during this very dynamic hormonal transition phase. Moreover, thanks to a broad recruitment campaign, a large sample consisting of 130 women was assessed in this study.

The present work provides an overview of physiological salivary E2 and P4 courses during the peripartum period in a sample of healthy women. Our results reveal great variations in the slope and fluctuations of E2 and P4, while the hormone ratio remains very stable. Health outcomes in the peripartum period should therefore be examined in light of these SS fluctuations and ratios. The group-based trajectory analysis revealed the presence of distinct SS courses throughout the pregnancy and postpartum period. In future research, these differences should be investigated in the light of epigenetic variations in SS receptors, as these can lead to altered receptor functioning and associated symptoms. Our results suggest that hormonal fluctuations at the end of pregnancy are a normal occurrence and might even constitute a protective factor for associated psychological symptoms and sleep disturbances in women.

## Data Availability

The raw data supporting the conclusions of this article will be made available by the authors, without undue reservation.

## Supplementary material

Supplementary Figure S1 **(A)** Salivary estradiol and **(B)** progesterone levels during pregnancy and postpartum (data within detection limits only /without imputated data). The grey dots and lines represent the mean and progressions of individual participants' hormone levels for the respective sex steroid and time point. The black dot indicates the mean and the black line the standard deviation of all participants for the respective time point. GW, gestational week; PP, postpartum.

Supplementary Figure S2 Hormone ratio of estradiol and progesterone (Log (P4/E2) = log (P4) − log (E2)) based on daily mean hormone levels (data within detection limits only/without imputated data). The grey dots and lines represent the individual participants' hormone ratio for the respective time point. The black dot indicates the mean and the black line the standard deviation of all participants for the respective time point. GW, gestational week; PP, postpartum.

Supplementary Figure S3 Fluctuations in **(A)** estradiol and **(B)** progesterone levels during pregnancy and postpartum (data within detection limits only /without imputated data). Fluctuations were operationalized as mean absolute successive difference (MASD). The grey dots and lines represent the mean and progression of individual participants' fluctuations (operationalized as MASD) for the respective sex steroid. The black dot indicates the mean and the black line the standard deviation of all participants for the respective time point. GW, gestational week; MASD, mean absolute successive difference; PP, postpartum.
