# Supplementary figures and images for: Estradiol and progesterone from pregnancy to postpartum: a longitudinal latent class analysis
Source: Front Glob Womens Health. 2024 Oct 9;5:1428494. doi: 10.3389/fgwh.2024.1428494 (PMC11496150; doi:10.3389/fgwh.2024.1428494)

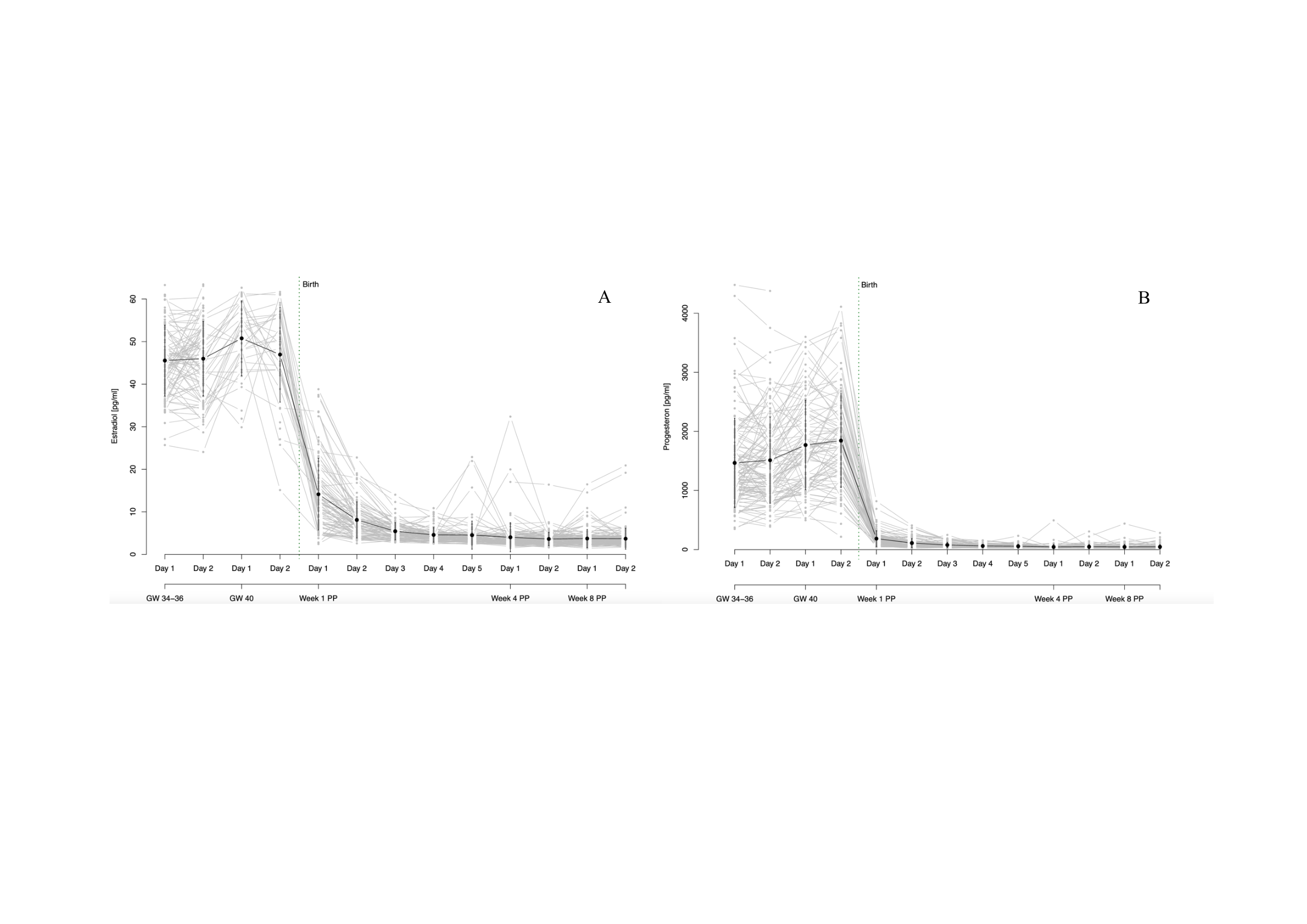

Supplement: Supplementary Figure S1 — (A) Salivary estradiol and (B) progesterone levels during pregnancy and postpartum (data within detection limits only /without imputated data). The grey dots and lines represent the mean and progressions of individual participants' hormone levels for the respective sex steroid and time point. The black dot indicates the mean and the black line the standard deviation of all participants for the respective time point. GW, gestational week; PP, postpartum. [file Image1.tiff]

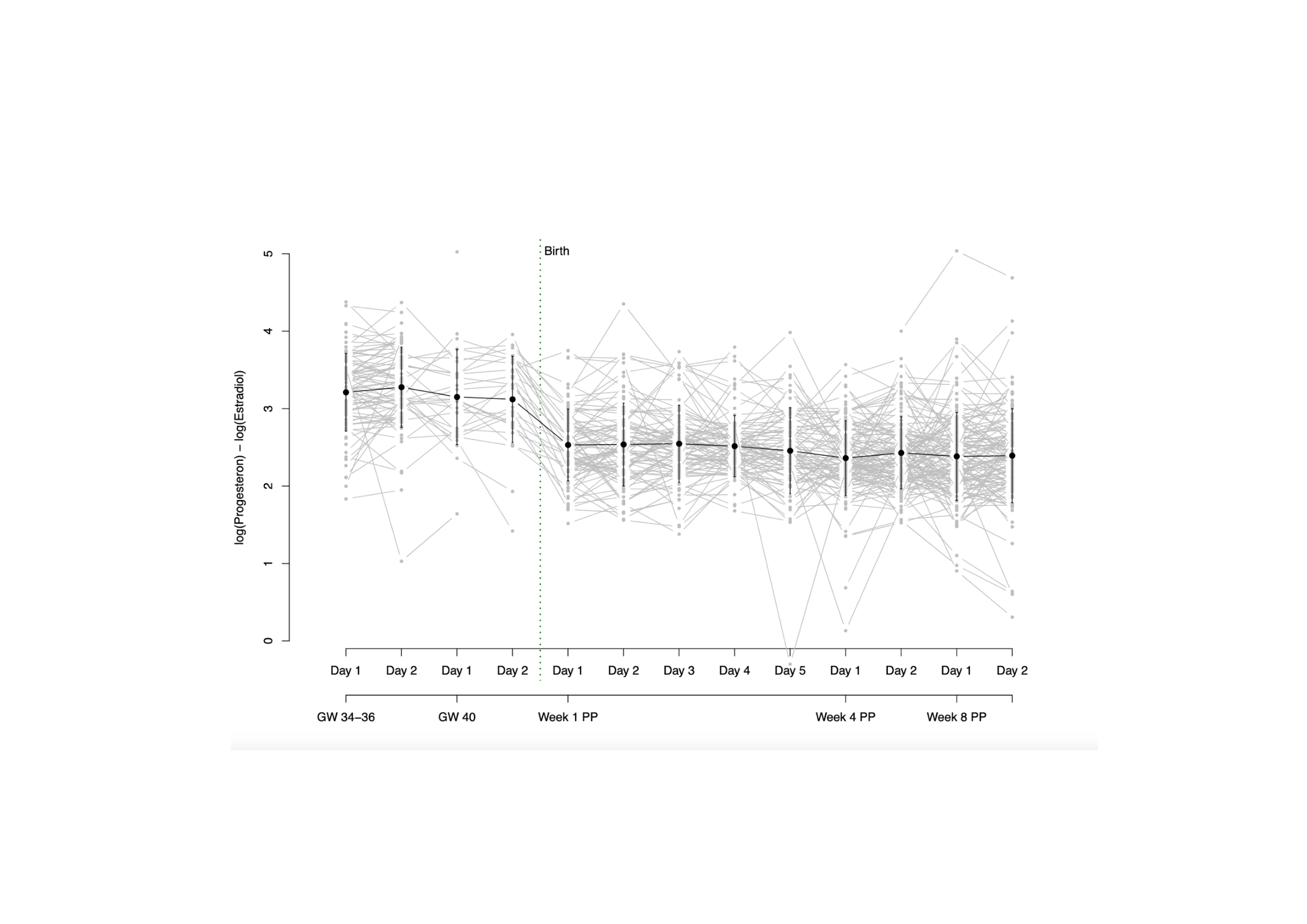

Supplement: Supplementary Figure S2 — Hormone ratio of estradiol and progesterone (Log (P4/E2) = log (P4) − log (E2)) based on daily mean hormone levels (data within detection limits only/without imputated data). The grey dots and lines represent the individual participants' hormone ratio for the respective time point. The black dot indicates the mean and the black line the standard deviation of all participants for the respective time point. GW, gestational week; PP, postpartum. [file Image2.tiff]

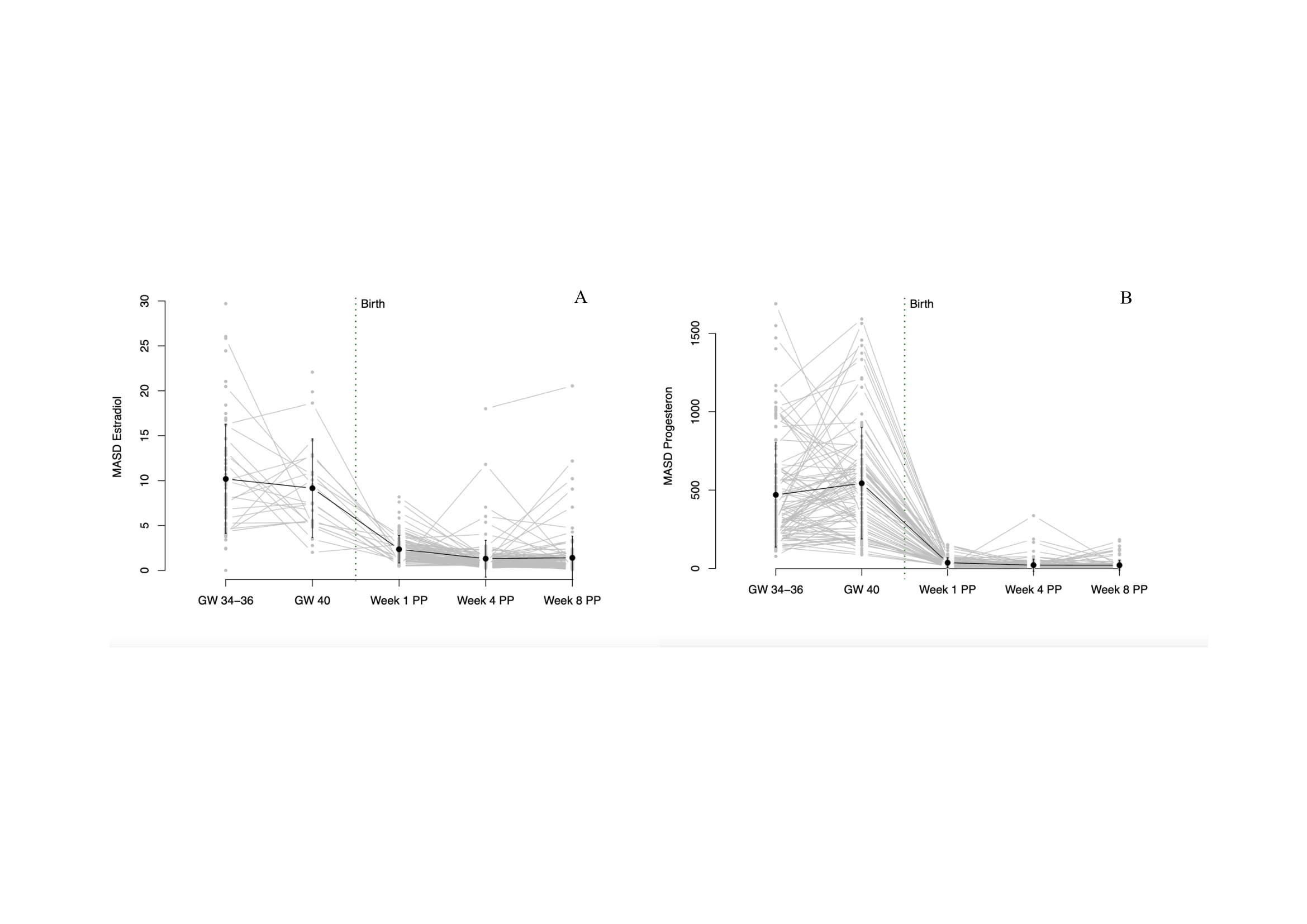

Supplement: Supplementary Figure S3 — Fluctuations in (A) estradiol and (B) progesterone levels during pregnancy and postpartum (data within detection limits only /without imputated data). Fluctuations were operationalized as mean absolute successive difference (MASD). The grey dots and lines represent the mean and progression of individual participants' fluctuations (operationalized as MASD) for the respective sex steroid. The black dot indicates the mean and the black line the standard deviation of all participants for the respective time point. GW, gestational week; MASD, mean absolute successive difference; PP, postpartum. [file Image3.tiff]
